# Sacral agenesis combined with spinopelvic dissociation: A case report and literature review

**DOI:** 10.1097/MD.0000000000012162

**Published:** 2018-09-14

**Authors:** Haiping Zhang, Hua Guo, Simin He, Hua Hui, Dingjun Hao

**Affiliations:** Department of Spine Surgery, Honghui Hospital, Xi’an Jiaotong University Health Science Center, Youyi east road 555#, Xi’an City, Shanxi Province, China.

**Keywords:** fusion, pelvic floating, sacral agenesis, spinopelvic dissociation

## Abstract

**Introduction:**

Sacral agenesis is a rare congenital disease with radiologic manifestation of sacrum deformity. Its clinical manifestations include spinopelvic instability due to sacroiliac joint deformity, spinal rotation, scoliosis, difficulties in walking, and claudication. Surgical intervention aims to prevent further deformity progression and to improve the patients’ walking function. It is challenging to achieve solid arthrodesis for this congenital disease, and fusion failure could aggravate deformity.

**Case presentation:**

We retrospectively studied one case of a 12-year-old girl with sacral agenesis combined with spinopelvic dissociation and spinal scoliosis. She was presented with intermittent lumbosacral pain and worsening walking instability. We reconstructed the posterior pelvic ring through 1 iliac screw implanted in the bilateral posterior superior iliac spine, and the preflexed connecting rod was tightly locked with bilateral screws through the opening at the right spinal process of S2. With this method, bilateral ilia and sacrum were integrated and hemipelvic floating could be corrected. Bone fusion was achieved between the bilateral ilia and the sacrum.

**Conclusion:**

Ilium-sacrum-ilium internal fixation and fusion for treating sacral agenesis combined with spinopelvic dissociation could achieve sacroiliac joint fusion. It is easy to perform and could cause little trauma while preserving the lumbar motion segment, which will provide new insight for treating sacral agenesis.

## 1 Introduction

Sacral agenesis is a rare congenital disease. The pathogenesis of sacral agenesis remains elusive. Some researchers suggest that maternal diabetes during pregnancy could cause sacral agenesis. Renshaw suggests that the sacral agenesis is due to the genetic mutation, which can cause formation dysfunction of the caudal myelin sheath and ventral spinal cord.^[1]^ Other scholars suggest that it is a sex-linked-dominant hereditary disease and autosomal-dominant disease.^[2,3]^ Patients with sacral agenesis often present with sacroiliac joint deformity, which further causes spinopelvic instability and spinopelvic dissociation. The affected side can also present with pelvic floating and combine with spine rotational deformity. Under such circumstances, spine surgeons often focus on the spinopelvic instability and pelvic tilt. And the spine fusion to the sacrum with further fixation can not only promote patients’ walking function, but also improve the secondary spinal rotation and scoliosis.^[4,5]^ However, due to the large defect between the hypoplastic sacrum and the ilium, there could be a considerable lack of bone cortex and a large amount of grafting bone would be needed. In such a situation, it is hard to achieve satisfactory bone fusion and is likely to yield surgical failure and aggravate deformity. A limited number of reports about treating sacral agenesis with spinosacral fusion suggest that these techniques could lead to great trauma, loss of activity due to lumbar fixation, a high incidence of infection, and complications such as nonunion.^[5–10]^

In the current study, we presented a case of sacral agenesis combined with spinopelvic dissociation, which was treated with posterior pelvic ring reconstruction and ilium-sacrum-ilium internal fixation and fusion. It could restore the biomechanical pillar structure between spine and pelvis, fuse sacroiliac joint, and achieve a sustained stability.

## 2 Case report

A 12-year-old female presented with intermittent lumbosacral pain for 2 years and worsening walking instability for half a year. This study was approved by the Institutional Review Broad of Honghui hospital, and the patient provided signed informed consent. She was admitted to the local hospital 2 years ago and underwent lumbar and pelvic frontal and lateral radiographs, which suggested sacral deformity. Over the past 6 months, she presented aggravating sedentary lumbar pain, gait instability, and lumbar tilt. She came to our institution for further medical advice. The patient was in good health before without any trauma or operation history. She was of full-term normal delivery without birth trauma or asphyxia, and her physical and mental developments were appropriate for her age.

**Figure 1:**
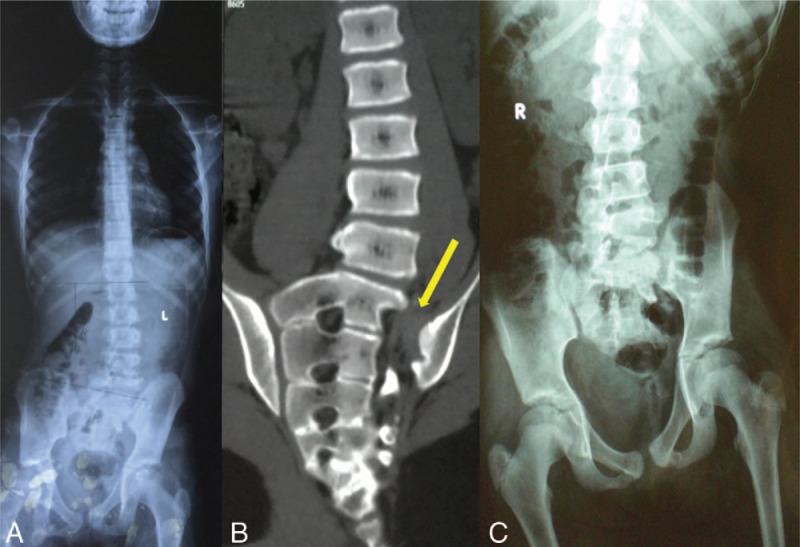
(A) X-ray of spine and pelvis in the supine position. (B) CT reconstruction revealed left sacral agenesis and displacement. (C) In the standing position, the patient's left sacrum was higher than the right with obvious pelvis tilt. CT = computed tomography.

Physical examination revealed that the patient had an abnormal gait and asymmetric pelvis, with the left iliac crest higher than the right. Left spinal rotation, scoliosis, and left buttock atrophy could be observed. Motion and sensation of limbs appeared intact, and bilateral physiologic reflexes were normal. X-ray of her spine suggested lumbar scoliosis from L1 to L5 and left sacrum agenesis with 10° Cobb angle (Fig. 1A). Computed tomography (CT) reconstruction revealed sacral wing agenesis from S1 to S5 with rotational displacement of left sacrum and sacroiliac dissociation (Fig. 1B). In the standing position, the patient's left sacrum was higher than the right, with obvious pelvis tilt (Fig. 1C). The diagnosis of congenital sacral agenesis combined with spinopelvic dissociation and spinal scoliosis was made. The preoperative scoliosis research society-22 (SRS-22) scores in terms of function/mobility, pain, appearance, and mental health were 10, 6, 13, and 6, respectively, with an average score of 6.2. The postoperative SRS-22 scores were 18, 14, 16, and 13, with an average score of 13.8.

## 3 Surgical intervention

**Figure 2:**
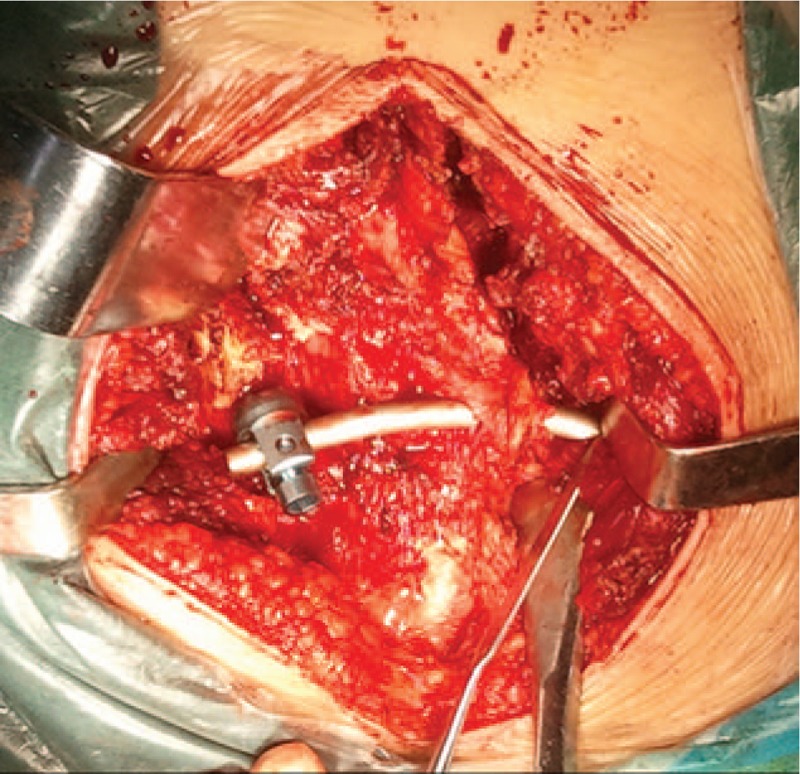
A connecting rod crossed the spinous process of S2 and ilium-sacrum-ilium internal fixation was achieved.

**Figure 3:**
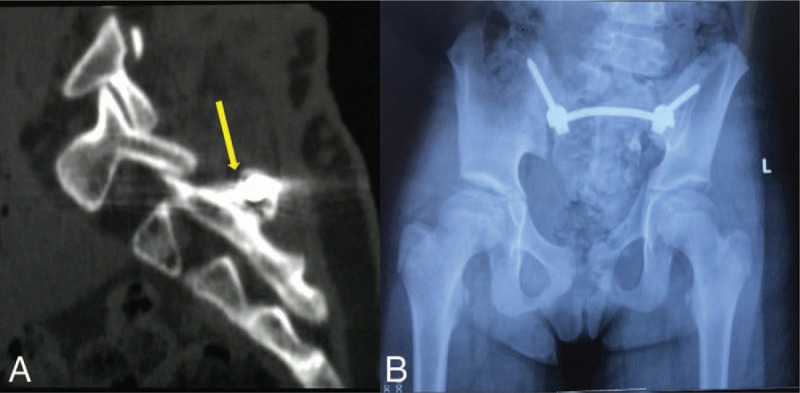
(A) Postoperative CT confirmed that the connecting rod passed through the spinous process completely. (B) X-ray suggested corrected pelvis floating in the standing position. CT = computed tomography.

**Figure 4:**
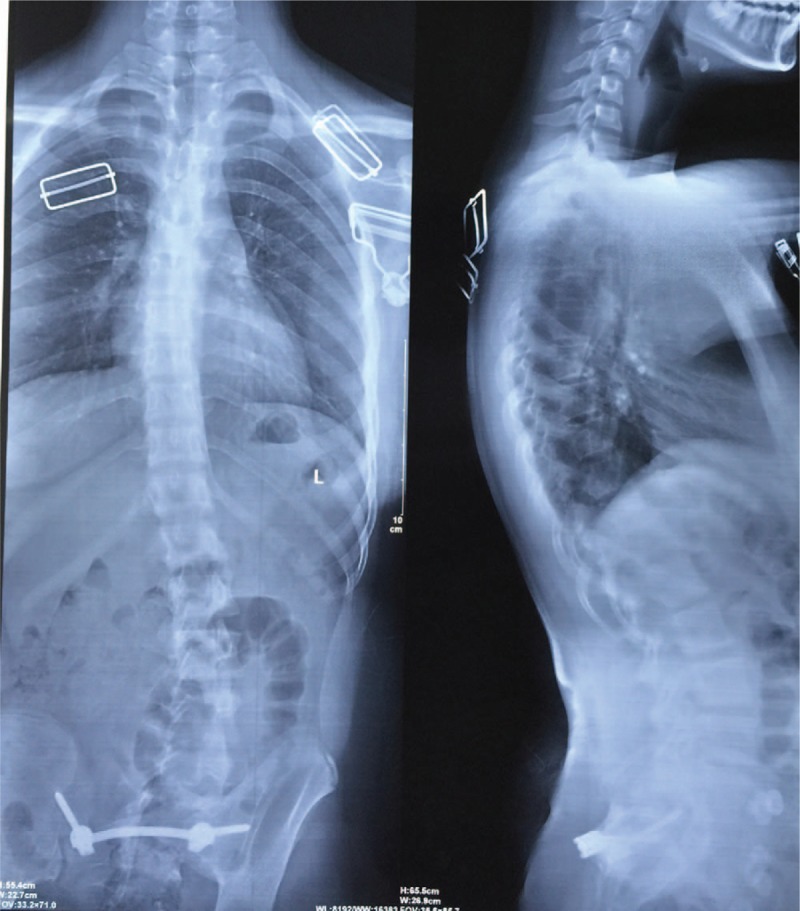
Shoulder, spine and pelvis balance was maintained at the fifth year of follow up.

**Figure 5:**
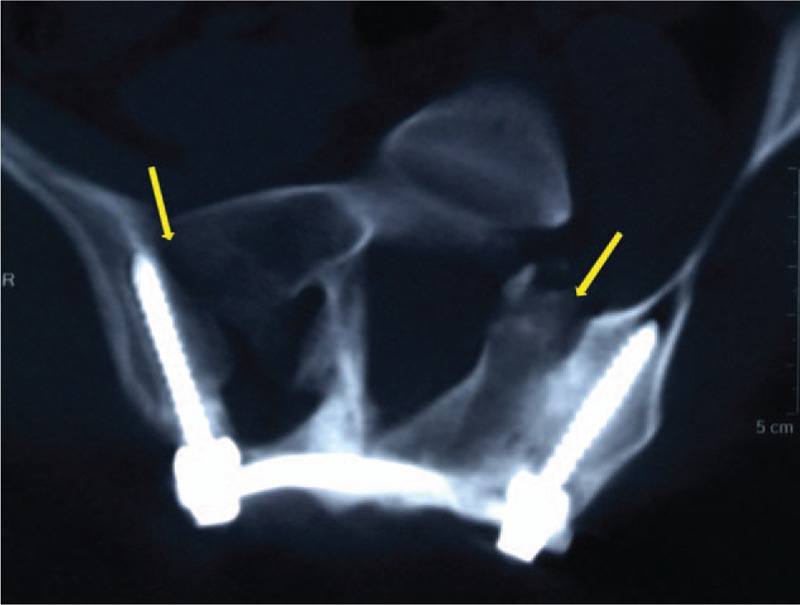
CT showed that complete joint fusion at bilateral sacroiliac joints at the fifth year of follow up. CT = computed tomography.

After general anesthesia, the patient was placed in the prone position, and a median incision of about 10 cm from L5 to S4 was made. After gradually exposing the spinous process, lamina, bilateral sacroiliac joints, and posterior superior iliac spine, dysplasia of the left sacral wing and left pedicle of S1-S4 could be observed. Iliac screws were placed bilaterally at the posterior superior iliac spine. An opening was made on the intact right spinous process of S2. Ilium-sacrum-ilium internal fixation was achieved, with the connecting rod crossing the spinous process of S2 (Fig. 2). C-arm fluoroscopy confirmed proper placement of internal fixation. The left outer table of ilium was trimmed in strips and placed at the left sacroiliac joint. After irrigation and placement of a drainage tube, the incision was closed layer by layer. Postoperative CT reconstruction confirmed that the connecting rod completely passed through the spinous process (Fig. 3A). X-ray suggested corrected pelvis floating in the standing position (Fig. 3B). Shoulder, spine, and pelvis balance was maintained at the fifth year of follow-up (Fig. 4). CT scanning revealed complete joint fusion at bilateral sacroiliac joints (Fig. 5).

## 4 Discussion

Sacral agenesis is a rare congenital malformation of sacrum. Its clinical manifestations vary among cases, and urinary incontinence is the most common symptom.^[11–14]^ Patients with sacral agenesis often present with spinopelvic instability, scoliosis, and neurological and visceral deformities. They may also present with no obvious appearance deformity or motion impairment. A diagnosis can usually be made according to x-ray results. Early in 1978, Renshaw et al classified sacral agenesis into 4 types: type I, partial or total unilateral sacral agenesis; type II, partial sacral agenesis with bilaterally symmetrical defect; type III, variable lumbar and total sacral agenesis, with the ilia articulating with the sides of lowest vertebra present; type IV, variable lumbar and total sacral agenesis, with the caudal endplate of the lowest vertebra resting above either fused ilia or iliac amphiarthrosis.^[1]^ In 1979, Stanley et al suggested classifying sacral anomalies into 3 types: agenetic type, total absence of vertebra with relative neurologic sparing, and a relatively low incidence of visceral congenital abnormalities; dysgenesis type, sacral defect that can be associated with hemivertebrae and butterfly vertebrae in the thoracolumbar spine and often complicated with visceral abnormalities, mild neurological deficit, and denervated bladder; dysraphic type, deficiencies of the neural arch leading to sacral spina bifida with meningocele or myelomeningocele, with no associated abnormalities except denervation of the lower urinary tract.^[15]^ And in 2002, Guille et al suggested a new classification of lumbosacral agenesis based on a retrospective study of 25 cases with lumbosacral agenesis: type A, there is either a slight gap between the ilia or the ilia are fused in the midline with 1 or more lumbar vertebrae absent, and the caudal aspect of the spine articulated with the pelvis in the midline, maintaining its vertical alignment; type B, the ilia are fused together with some of the lumbar vertebrae absent, and most caudal lumbar vertebra articulating with one of the ilia with the most caudal aspect of the spine shifting away from the midline; type C, a total agenesis of the lumbar spine with the ilia fused together, with a visible gap between the most caudal intact thoracic vertebra and the pelvis.^[13]^ This classification could predict ambulatory potential according to the deformity degree, which could help identify the proper treatment method.

Regardless of the classification methods, we suggest that whether operative treatment shall be taken for patients with sacral agenesis mainly depends on ambulatory potential and spinopelvic stability. Conservative treatment is preferred when the patient cannot walk without obvious pelvic tilt, skin ulcers, or evidence of progressive deformity aggravation. For patients with progressive spinopelvic deformity and obvious spinopelvic instability, especially when the patient can walk, early operative treatment is preferred. In the current study, walking imbalance was present in the 12-year-old female patient. After surgical intervention, the walking imbalance was notably improved in the 5-year follow-up. The aim of surgical intervention is to stop the progression of deformity, to correct walking imbalance, and to enhance spinopelvic stability. The essence of achieving spinopelvic stability for such congenital deformity is to achieve bone fusion. However, for sacral agenesis with large bone defect, it is challenging to achieve bone fusion, and failed fusion could lead to aggravated deformity and other complications. According to the limited number of studies on spinopelvic stability, we found that bone nonunion had a relatively high incidence.^[5,6,8]^ Yazici et al reported 3 cases with lumbosacral agenesis that underwent posterior lumbopelvic instrumentation and fixation with autogenic anterior tibial cortical structural graft for laminopelvic bridging and demineralized bone matrix for augmentation of osteoinduction.^[7]^ Successful fusion was achieved for all 3 patients, which allowed for sitting. It should be noted that the autogenic anterior tibial cortical structural graft could cause other diseases, and the donor tibia was secured with an intramedullary pin, which could cause further trauma to the patient.^[7]^ Ferland et al described a novel surgical technique that involved spinal instrumentation and correction of deformity with impaction of vascularized ribs from the spine to the iliac crest.^[10]^ Six patients with sacral agenesis achieved solid spinal and spinopelvic fusion. However, the infection rate was up to 50%, and 4 patients underwent revision surgeries.^[10]^

Resection of sacral chordoma will cause iatrogenic injury equivalent to type II Renshaw defect and a subsequent spinopelvic stability problem. Our novel technique could solve this issue by applying a pedicle screw fixation system to reconstruct the posterior pelvic ring and stabilize the sacroiliac joint. After the operation, pelvic obliquity was maintained and pelvic asymmetry was improved. At the fifth year of the follow-up, lumbar rotational deformity presented no progression. CT scanning further revealed 3 interesting findings. First, massive callous formation was observed at the left sacral lamina, which would yield firm bone fusion. And the callus formation was more obvious at the defect side of the sacrum than the intact part. From the aspect of anatomy, the connecting rod crossing the spinous process of S2 could conduct stress to the bilateral lower limbs, mimicking the physiologic spinopelvic stress conduction. In that process, screw fretting could occur, which could stimulate callus growth and accelerate fracture healing.^[16]^ However, the underlying mechanism of how mechanical environment could affect fracture healing remains largely unknown. As the right part of the sacrum was intact and had less stress burden than the left side, the screw at the defect part bore more stress than the right side, and thus more callus formation could be observed at the defect part. Several studies have suggested that proper stress could stimulate fracture healing,^[17,18]^ and one focus of how stress modulate fracture healing is the stimulus of stress on bone tissues. Some scholars have suggested that the mechanoreceptors on the surface of bone cells could receive the surrounding stress stimulus and react accordingly.^[19,20]^ While some have scholars suggested that when bone cells are under stress, the flow of intracellular matrix and cytoplasm could cause local potential change, namely, the piezoelectric effect of bone.^[21]^ Regardless of how stress affects bone tissues, bone tissues could ”sense” the mechanical changes and change their own metabolism as a feedback. In our method, we speculate that callus formation could be due to the axial force that stimulates osteoblast proliferation, which could solve problems such as the source of grafting bone and the complications of bone grafting. Second, the ilium-sacrum-ilium internal fixation and fusion system could correct the pelvic floating position instantly, and the patient presented marked improvement in walking after operation. Thirdly, during the 5-year follow-up, we noticed that this technique blocked left rotation of the affected sacrum and stopped the progression of lumbar rotational deformity. Notable improvement in walking balance could be observed as well.

There are several limitations to the current study. First, we only reported 1 case of sacral agenesis combined with spinopelvic dissociation, and our experienced might not be suitable for all cases of sacral agenesis combined with spinopelvic dissociation. Also, the underlying mechanism of the satisfactory bone fusion observed in this case remains elusive.

In conclusion, this study provided ilium-sacrum-ilium internal fixation and fusion for treating sacral agenesis combined with spinopelvic dissociation. It is easy to perform and could cause little trauma while preserving the lumbar motion segment, which will provide new insight for treating sacral agenesis.

## Author contributions

**Conceptualization:** Hua Guo.

**Data curation:** Hua Guo, Hua Hui.

**Formal analysis:** Hua Hui.

**Investigation:** Haiping Zhang, Hua Guo.

**Methodology:** Haiping Zhang, Simin He, Hua Hui.

**Resources:** dingjun hao.

**Software:** Hua Hui.

**Supervision:** dingjun hao.

**Validation:** Simin He.

**Visualization:** Simin He.

**Writing – original draft:** Haiping Zhang.

**Writing – review & editing:** dingjun hao.
